# Svalbard-barents ice sheet instability enhanced delivery of reactive iron to the ocean

**DOI:** 10.1038/s41467-026-75133-2

**Published:** 2026-07-01

**Authors:** Allyson Tessin, Christian März, Johan C. Faust, Philipp Böning, Matthias Forwick, Rachael Gray, Jens Matthiessen, Anke Neumann, Matthew O’Regan, Bernhard Schnetger, Christoph Vogt

**Affiliations:** 1 Department of Earth Sciences, Kent State University, Kent, OH USA; 2 School of Earth and Environment, University of Leeds, Leeds, UK; 3 Institute for Geosciences, University of Bonn, Bonn, Germany; 4 Center for Marine Environmental Sciences (MARUM), University of Bremen, Bremen, Germany; 5 Institute for Chemistry and Biology of the Marine Environment, University of Oldenburg, Oldenburg, Germany; 6 Department of Geosciences, UiT The Arctic University of Norway, Tromsø, Norway; 7 Alfred Wegener Polar Institute, Helmholtz Centre for Polar and Marine Research, Bremerhaven, Germany; 8 PSI Center for Nuclear Engineering and Sciences, Paul Scherrer Institut, Villigen, Switzerland; 9 Department of Geological Sciences, Stockholm University, Stockholm, Sweden; 10 Faculty of Geosciences, Crystallography & Geomaterials Research, University of Bremen, Bremen, Germany

**Keywords:** Element cycles, Palaeoceanography

## Abstract

Iron (Fe) plays a key role in marine environments as a bio-limiting nutrient and a control on sedimentary burial of carbon and other nutrients. Sensitive glacial systems provide an important delivery mechanism for Fe to the ocean, but it is unknown how these glacial sources will change as ice retreats. Sedimentary records from the Yermak Plateau, proximal to the Svalbard-Barents Ice Sheet provide an opportunity to investigate how past glacial instability may have affected bioavailable iron delivery. Here we find that over the past 135 ka, pulsed reactive Fe export to the Yermak Plateau occurred at least 5 times, during intervals of ice sheet retreat or advance. Fe-rich layers preserve high contents of reactive Fe phases, some of which were likely bioavailable at the time of deposition. Glacial Fe from Svalbard may have been delivered either directly by meltwater plumes or through remobilization of Fe deposited in fjord and shelf sediments. The delivery of reactive Fe hundreds of kilometers offshore during these events indicates that ice sheet instability in modern settings could perturb high latitude nutrient cycles.

## Abstract (web summary)

Multiple iron delivery events to the Yermak Plateau associated with episodes of ice sheet retreat and advance over the past 135 thousand years indicate that ice sheet instability in modern settings could perturb high latitude nutrient cycles.

## Introduction

Marine primary productivity plays an important role in the global carbon cycle and can regulate atmospheric CO_2_ through changes in the biological pump^1^. The importance of the micronutrient Fe in the regulation of primary productivity is identified both within high nutrient, low chlorophyll regions (HNLCs)^2,3^, and in nitrate limited regions, where Fe availability can regulate nitrogen fixation^4–6^. Initial study of Fe limitations focused on the importance of dust-borne Fe^7,8^, however, the influence of other Fe sources, including benthic remobilization^9,10^ and hydrothermal activity^11–13^, has proved to be important, particularly at high latitudes^14^.

Increasing evidence suggests that glacial sources including icebergs^15,16^ and meltwater^17–19^ are important contributors of bioavailable Fe to high latitude oceans. Weathering processes in sub- and proglacial environments enrich meltwaters with Fe^20,21^. However, meltwater in many glaciated regions must first pass through estuaries before entering the shelf and open ocean, where Fe may be lost and intermittently trapped due to flocculation, thereby inhibiting delivery to the ocean. Studies investigating the behavior of dissolved Fe within fjords indicate that 80–98% of Fe is sequestered in fjord sediments^22–24^, calling into question whether meltwater-derived Fe plays a significant role in marine productivity beyond coastal environments. Conversely, the accumulation of Fe-rich sediments within these estuarine environments could provide a source of Fe offshore if this sediment is remobilized.

While evidence for Fe limitations is abundant in the southern high latitude oceans^25^, the Arctic Ocean is generally thought to be light-limited^26^ and nitrate-limited when nutrient limitations occur^27^. However, there is evidence that Fe limitations already exist within the Arctic^28,29^. Specifically, large under-ice blooms in the Nansen basin are Fe-limited, and Fe may limit primary productivity in the Barents Sea^30^. In the modern ocean, nitrate-limited regions are largely controlled by iron supply^6^ because Fe is required for the nitrogenase enzyme used by diazotrophs to fix nitrogen, indicating that Fe supply will be important in the Arctic under either Fe- or nitrate-limited conditions.

High latitude regions are currently undergoing significant perturbations in response to anthropogenic climate change. Mass loss from the Greenland and the Western Antarctic ice sheets is increasing^31–33^. As these ice sheets continue to rapidly melt, an improved understanding of the fate of glacially-derived Fe is required to evaluate how modern ice sheet instability will affect nutrient availability and primary productivity.

**Fig. 1:**
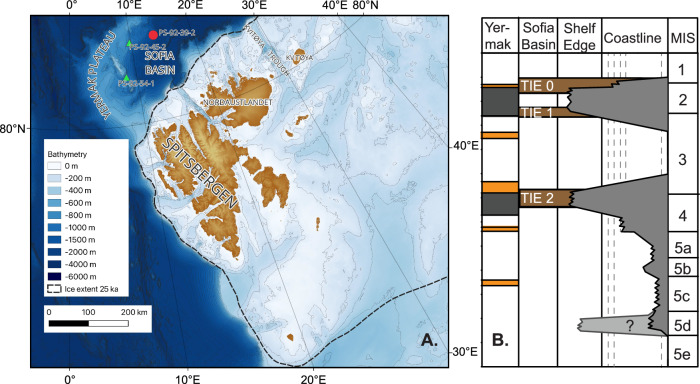
Study Area and geologic context. **A** Map of the study area. The red circle denotes the core location for PS92/39-2, and the green triangles denote the locations for PS92/45-2 and PS92/54-1. Bathymetry is from IBCAO v. 4^89^. The LGM extent of the Svalbard-Barents Ice Sheet (SBIS) is denoted by a dashed black line^34^. **B** Regional glacial and sedimentological events. Yermak Plateau includes general sedimentology of core PS92/39-2 with gray bars indicating two distinct intervals of gray clay and orange bars indicating laminated layers that are generally orange-brown in color^90^. Terrigenous input events (TIEs) are denoted as observed in the Sofia Basin^70^. Glacial extent of the SBIS is indicated in lighter gray^91^.

In this study, we produce records from core PS92/39-2 (1464 m water depth) from the Yermak Plateau (~300 km north of the Spitsbergen coast) to investigate if transport of reactive Fe away from the coast and onto the Yermak Plateau changed during past climate fluctuations (Fig. 1A). Additional results from two other gravity cores, PS92/45-2 (915 m water depth) and PS92/54-1 (1145 m water depth), are included. For our purposes, reactive Fe refers to Fe phases that are susceptible to biogeochemical transformation, a portion of which were likely bioavailable at the time of deposition. These sites are proximal to the past Svalbard-Barents Ice Sheet (SBIS), which experienced repeated phases of glacial advance and retreat during the late Quaternary^34^. During the last glacial maximum, the SBIS was marine terminating before retreating from the shelf edge since 19 ka^34^. The sedimentary record presented here covers the past 135 ka and includes glacial-interglacial transitions, records intervals associated with millennial-scale climate instability, and provides context for the biogeochemical implications of future ice retreat in Greenland and West Antarctica.

## Results

**Fig. 2:**
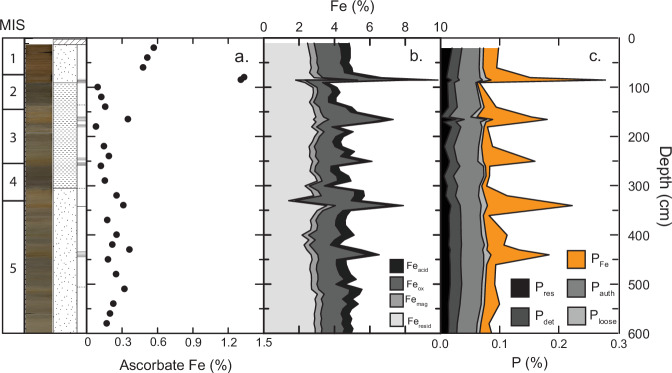
Geochemical results from core PS92/39-2 with Marine Isotope Stages (MIS), core photo, and stratigraphic column. **a** Downcore record of ascorbate extractable Fe **b** Fe speciation results from sequential chemical extractions, and **c** P speciation from chemical sequential extractions. Fe extractions include acid-soluble Fe_acid_ (0.5 M HCl), Fe_ox_ (oxyhydr)oxides (sodium dithionite), and magnetite Fe_mag_ (ammonium oxalate). P extractions include loosely bound P_loose_ (MgCl_2_), Fe-bound P_Fe_ (sodium dithionite), authigenic apatite (sodium acetate; P_auth_), detrital apatite (HCl; P_det_), and residual P_res_ (including organic P). Details of extractions can be found in the “Methods” section. MIS - Marine Isotope Stages with tie points^36^.

**Fig. 3:**
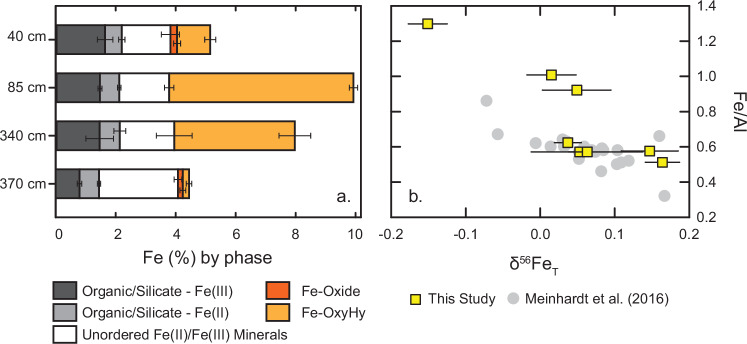
Detailed Fe geochemistry from core PS92/39-2. **a** Solid Fe speciation determined by Mössbauer spectroscopy for samples at 40, 85, 340, and 370 cm converted to absolute content using total Fe content determined by XRF. Errors are adjusted based on the Fe concentration. **b** Total Fe isotope (δ^56^Fe_T_) measurements (yellow squares). Previously published Fe isotope values from the Arctic Ocean are plotted as gray circles^68^.

To investigate changes in biogeochemically reactive Fe since the last interglacial, we focus on the upper 6 m of core PS92/39-2, which covers the last 130 ka. The age model for PS92/39-2 was developed through correlation of environmental rock magnetic parameters to the global benthic δ^18^O record, and supported by radiocarbon dating, stable isotope analysis and amino acid racemization of planktic and benthic foraminifera^35,36^ (Fig. 1B). Total iron (Fe_T_) contents in PS92/39-2 range between 3.49 and 9.85%, with peaks occurring at 85, 165, 250, 340, and 440 cm with smaller peaks at 280 and 510 cm (Fig. 2). Chemical Fe speciation data indicate that reactive Fe phases (acid-extractable, crystalline Fe oxides, and magnetite) are relatively more abundant in Fe_T_-rich layers, with reactive phases representing 38 to 84% of Fe_T_ (Fig. 2). Ascorbate extractable Fe ranges from 0.48–1.33% in the upper meter of the core, with the highest values found in the Fe-rich layer at 80–85 cm (Fig. 3). Below 1 m, ascorbate extractable Fe contents range between 0.08 and 0.36%, with higher contents corresponding to higher Fe_T_ contents. Based on Mössbauer spectroscopy, Fe (oxyhydr)oxides increase in samples with elevated Fe_T_ contents (51–62% of the Fe pool, as compared to 4–23%; yellow bars in Fig. 3B). This component exhibits Mössbauer parameters (Table S1) consistent with ferrihydrite and/or goethite, with the latter being poorly ordered and/or of small size as indicated by its magnetic ordering occurring well below room temperature^37^. We consider these poorly ordered Fe (oxyhydr)oxide(s), together with the incompletely ordered component we observed at 4 K (white bars in Fig. 3), as reactive Fe phases^38^. The δ^56^Fe_T_ values range between -0.151 and 0.164‰ (Fig. 3). In general, the δ^56^Fe_T_ decreases with increasing Fe_T_ content.

**Fig. 4:**
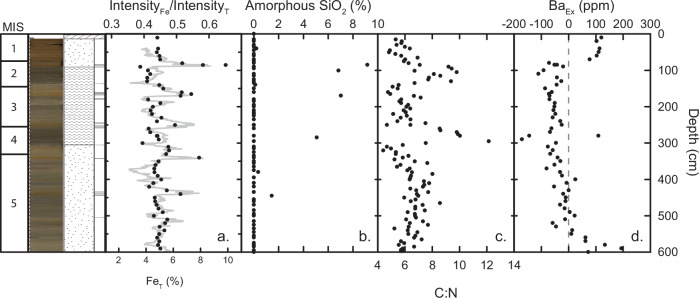
Productivity and organic matter indicators with Marine Isotope Stages (MIS), core photo, and stratigraphic column. **a** Total Fe contents from qualitative scanning XRF (intensity ratio) and discrete quantitative XRF (%) compared to indicators of shifts in primary productivity in core PS92/39-2: **b** Amorphous silica content from XRD analysis, **c** Previously published total organic carbon to total nitrogen (C: N) ratios^90^, and **d** Barium excess (Ba_Ex_) content from quantitative XRF.

Total P contents in the record range between 0.07 and 0.28% (Fig. 2). Within the high Fe_T_ samples, the Fe-bound P pool increases up to three-fold relative to other samples. Excluding Fe-bound P, the contents of the other phases are relatively consistent throughout the record, ranging between 0.06 and 0.09%. Where total P is less than 0.1%, Fe-associated P is between 6 and 26% of total P, while Fe-associated P makes up 50–70% of total P where total P is >0.15%. Excess barium (Ba_Ex_) contents range between −171 and 194 ppm, with values being generally elevated during the Holocene and the last interglacial (Marine Isotope Stage (MIS) 5e) with the exception of an enrichment at 280 cm (Fig. 4). Amorphous Si content is near or below detection limit with the exception of five samples, in which amorphous Si content ranges from 1.4–9.2% (Fig. 4).

## Discussion

The Fe-rich sediment layers found across the Yermak Plateau are interpreted as primary depositional features rather than post-depositional oxidation events, as they represent distinct, laminated sediment layers. In addition, their Fe content is asymmetrical, with the highest Fe values observed at the base followed by a gradual decrease into overlying sediments (Fig. 2). Laminated sedimentation and a lack of bioturbation within these layers^39^ suggests oxygen conditions were relatively lower during their deposition as compared to surrounding sediments, while a buried redoxcline with precipitated, oxidized Fe requires oxygenated sediments overlying more anoxic reducing sediments. Sedimentary iron Fe content driven by post-deposition oxidation would also lack the systematic asymmetry, or display a distinct upper boundary with high Fe contents as porewaters interacted with oxygen. Within the core, there are also distinct Mn-rich layers, which appear to be buried diagenetic fronts, which are not coincident with the Fe-rich layers, arguing against a diagenetic co-enrichment of Fe and Mn (oxyhydr)oxides (Supplementary Figs. S2, S3).

Layers of Fe_T_-rich sediments of between 8–10% are found within three cores across the Yermak Plateau (Supplementary Fig. S4), despite different water depths and oceanographic settings, supporting that these layers represent intervals of changing sediment dynamics across the region. These layers are on average 3 cm thick and represent an increase of 4% Fe over baseline Fe contents. If we assume that the entire area between our three sites (~7000 km^3^) experienced this amount of Fe delivery, around 13.4 Tg of excess particulate Fe input would have been deposited across this region. This amount of Fe is equivalent to ~10 years of the modern particulate Fe input into the oceans from Greenland or >100 years of the average modern input from Antarctica.

Controls on the distribution of Fe on high latitude marine margins include the composition of the bedrock and hydrological conditions^40^. The main proximal source of Fe to the Yermak Plateau is the Svalbard archipelago, although more distal material could be transported via ice rafting from the Siberian shelf by the Transpolar Drift. Northern Svalbard bedrock geology is diverse, ranging from Proterozoic metamorphic rocks to Paleogene sedimentary rocks^41^. The presence of reddish, Fe-oxide-rich sediments is usually linked to the Devonian Old Red Sandstone, which outcrops in north central Svalbard and is a dominant bedrock type in multiple watersheds that drain to the west and the north. Weathering of the Devonian Old Red Sandstone delivers pink-colored sediments and reactive Fe to fjords on Svalbard^40^ and periodically to the western shelf^42,43^ and the Barents Sea^43^. The presence of tidewater or marine-terminating glaciers, as compared to land-terminating glaciers, was likely an additional key control on the delivery of Fe^40,44^, because reactive Fe generated within the subglacial environment can directly enter marine waters and not be trapped within oxic terrestrial environments.

In general, Fe(II) is regarded as more bioavailable than Fe(III)^45,46^ and poorly crystalline Fe (III) (oxyhydr)oxides are considered to be more bioavailable than more crystalline minerals^47^, although evaluating the bioavailability of different Fe phases is complicated. Determining the bioavailability of Fe preserved within the marine sedimentary record is even more complex, because Fe undergoes early diagenetic alteration within sediments, including aging of Fe (oxyhyrdr)oxides to more crystalline minerals. Despite these complications, the abundance of reactive phases can be used to infer the degree to which the bioavailable pool of sedimentary Fe changed through time.

The Fe speciation data (both Mössbauer and operationally defined chemical extractions) indicate that Fe_T_-rich layers contain a higher fraction of certain reactive Fe phases relative to Fe_T_-poor layers (Fig. 3). These two techniques target different pools of Fe chemical extractions, and while the results are not directly comparable, they provide complementary information about Fe phases. Mössbauer results indicate that a larger amount of poorly ordered and amorphous Fe oxyhydroxides exists in Fe_T_-rich layers compared to Fe_T_-poor layers, most likely in the mineral form of goethite. Sequential chemical Fe extractions (Fig. 2) indicate that the Fe_T_-rich layers contain more reactive Fe minerals. Notably, all samples from the record contain large amounts of reactive Fe (38–84% of Fe_T_ in all samples). For comparison, 38% of Fe_T_ is considered the maximum portion of highly reactive Fe expected under oxic conditions in modern and Phanerozoic marine sediments^48^, which highlights that this region consistently contains atypically reactive Fe-rich sediments given the oxic depositional environment.

To further investigate whether labile nano-oxyhydroxides, specifically 2-line ferrihydrite, were present, an ascorbate extraction was used on a separate sediment split^49^. While all samples include a non-zero contribution of ascorbate extractable Fe, large enrichments (>0.5%) only occur in the upper meter of the section within Fe_T_-rich beds (especially at 85 cm), supporting that some amount of ferrihydrite persists within sediments, even if goethite is the dominant oxyhydroxide form. These Fe_asc_ contents exceed Fe_asc_ found within most glacial source materials on Svalbard and the Devonian bedrock^50,51^, which demonstrates the need for a mechanism that increased Fe reactivity between the glacial source and the Yermak Plateau. Similarly high Fe_asc_ contents (up to 1 wt.%) have been observed in fjord sediments^52^, which suggests that remobilization of fjord or shelf sediments could result in Fe_asc_-rich sediment deposition on the plateau.

We speculate that the absence of large downcore enrichments in Fe_asc_ is due to aging of nano-oxyhydroxide minerals into more crystalline phases with time, and not a result of less delivery of these phases earlier in the record. However, it should be noted that while the Fe_asc_ data suggests a significant decline in ferrihydrite contents, the Mössbauer results do not show as significant a decline in nano-oxyhydroxide minerals between 85 and 340 cm. This indicates that the total pool of nano-oxyhydroxide minerals is not smaller within deeper Fe_T_-rich layers, but the balance between specific phases does change. This aging of ferrihydrite to goethite is still slower than would be anticipated, suggesting that sedimentary conditions inhibit this process, potentially due to adsorption/complexation of ferrihydrite with silica, phosphate, and/or organic carbon^53^. Mössbauer results also suggest the presence of a very reactive unordered oxyhydroxide phase in Fe_T_-poor samples (370 cm in particular) that is not targeted by any of our chemical extractions. Mössbauer results record a significant pool of Fe in silicate and organic phases that is relatively stable throughout the record (Fig. 3), indicating that these are not sensitive to changes in glacial activity.

The reactivity of Fe within Fe-rich layers is further supported by P speciation results (Fig. 2). Fe-rich layers are associated with higher P contents, which are driven by a large increase in Fe-bound P. The P sequestered by Fe oxides was likely released by metal oxide reduction and/or organic matter remineralization. The role of reactive, oxidized Fe phases in P retention is well-documented across marine and coastal settings^54^. These results highlight that periodic input of reactive Fe can trap P in the water column, impacting other nutrient cycles.

Additional geochemical evidence suggests that increased Fe delivery to the Yermak Plateau fueled increased primary productivity (Fig. 4). The Fe-rich layers at 85, 165, 280, and 440 cm are associated with four of the five peaks in amorphous silica in the record, indicating increased delivery of biogenic opal to the seafloor. Additionally, after Fe peaks at 85, 165, 240, and 340 cm, C:N ratios decrease, indicating a shift to more marine algal productivity. Algal organic matter has a C:N ratio of 4–10, while terrestrial organic matter has a ratio of > 20^55^. While its interpretation can be complex, especially in the Arctic^56^, Ba content is often used as a proxy for carbon export from the surface ocean^57^, especially in sites >1000 m of water depth. After Fe peaks at 85, 165, and 240 cm, Ba contents locally increase and at 280 cm, a smaller Fe peak is associated with a large increase in Ba contents, supporting a shift towards more export productivity during or after Fe delivery events.

Multiple physical mechanisms could deliver glacial Fe to the Yermak Plateau, including ice rafting, which is a key mechanism in the Fe-limited Southern Ocean^58,59^. However, the Fe_T_-rich layers within the Yermak Plateau sedimentary record occur during minima of ice rafted debris (Supplementary Fig. S1). Therefore, icebergs were unlikely to be a primary pathway of Fe delivery.

Meltwater delivered Fe has been observed in high latitude regions^60–64^ and is another possible delivery mechanism to the Yermak Plateau. The association between Fe-rich sediments and laminations supports a meltwater origin, as laminations are a characteristic feature of glacimarine sedimentation induced by suspension settling from meltwater plumes^65^. Meltwater controls on Fe mineral deposition are significant enough that sedimentary Fe (oxyhydr)oxides have even been proposed as a proxy for reconstructing glacial mass loss^66^. However, meltwater from Svalbard must first transit through fjord systems, potentially resulting in significant Fe loss due to the salinity gradient^23,24^. While this process may have occurred during deposition of PS92/39-2 sediments, Fe deposited within fjord and shelf sediments is not necessarily a permanent sink, and ongoing benthic recycling in Svalbard fjords has been shown to facilitate the transport of glacially derived dissolved Fe across fjords and potentially into adjacent continental shelf regions^40,51^.

Physical remobilization of Fe deposited within more proximal environments during intervals of ice sheet advance and retreat is supported by the high Fe_asc_ contents, which are unlikely to have been directly deposited from meltwater. Fe_T_ isotope results (Fig. 3) support that there was an intermediate step of deposition within fjord and shelf sediments, followed by remobilization to coastal waters and beyond. Dissimilatory Fe reduction occurs in organic-rich Arctic shelf sediments, resulting in non-lithogenic Fe (oxyhydr)oxide enrichments at the sediment surface^67^. Lower δ^56^Fe values observed further into the Arctic basins has been interpreted as a basin-ward transport of these shelf sediments^68^. Although the range of isotope values is small, Fe_T_-rich sediment layers on the Yermak Plateau are associated with increased accumulation of ^54^Fe compared to nearby Fe-poor layers, suggesting the Fe may have undergone redox cycling within estuarine or shelf environments prior to delivery to the Yermak Plateau. Fe-rich sediments sourced from benthic remobilization could have then been entrained in the West Spitsbergen Current (Fig. 1) and transported north and eventually east to the Yermak Plateau.

The timing of Fe_T_-rich deposition events provides important insight into the relationship between Fe input to the Yermak Plateau, glacial instability, and climate. The timing of Fe-enrichments corresponds to changes in ice sheet extent, with a particular sensitivity to advance and retreat near the shelf break (Fig. 1b). Two of the Fe_T_-rich layers occur within laminated sediments, which directly overlie distinct dark gray layers identified as terrigenous input layers interpreted as periods of intensive glacial activity on Svalbard^69,70^. The Fe_T_-rich layer at 85 cm was deposited during Termination I when the Svalbard Barents Sea ice sheet was retreating to the coastline of northern Svalbard. The timing of the Fe_T_-rich, laminated deposition is broadly consistent with Bølling age (~14.5 ka) laminated beds that were deposited on the western^71–74^ and eastern^75^ margins of Svalbard during Meltwater Pulse 1a (14.6–14.3 ka)^76^, and during deglaciation on the northern margin of Svalbard^66^. The Fe_T_-rich layer at 250 cm occurs at or near the MIS 4/3 transition and was likely deposited during retreat of the MIS 4 ice sheet. Previous research on the Yermak Plateau indicated that conditions during the MIS 4/3 transition were similar to Termination I (MIS 2/1 transition), which suggests that times of rapid ice-sheet retreat are associated with increased delivery of bioavailable Fe to the open ocean.

In addition to the layers at 85 cm and 250 cm, Fe_T_-rich layers in core PS92/39-2 occur during relatively warmer climate conditions during MIS 3 and 5, implying that large-scale ice-sheet retreat is not the only time when bioavailable Fe delivery to the open ocean is enhanced. Previous work investigating meltwater pulses in the Fram Strait during MIS 3 highlighted that the Svalbard Barents ice sheet was sensitive to millennial-scale warming events^42^. These meltwater events were identified based on deposition of fine-grained sediments sourced from Svalbard Devonian red beds that were similar in nature to the laminated sediments of Bølling age. Our results, paired with this previous work, highlight that the climate-driven instability of the Svalbard-Barents Ice Sheet during MIS 3 and 5 led to input of Fe_T_-rich material to the shelves and past the shelf break, where it may have fueled enhanced primary productivity. Age constraints from the presented records and fragmentary glacial records from Svalbard preclude a detailed analysis of the exact timing of these events. However, Fe deposition peaks occur within both MIS 3 and 5 but not during the Holocene (Fig. 2) as supported by a lack of Fe peaks in published multicore data^77^, which suggests that significant transport of Fe-rich material requires the presence of glaciers that terminate closer to the ocean than those that exist today. But importantly, the lack of evidence for ice sheets extending to the shelf edge during MIS 3 and 5 implies that ice sheets did not need to be as large as those which existed during glacial maximum conditions.

Our sedimentary and geochemical records from the Yermak Plateau demonstrate that ice sheet instability can significantly perturb the Fe cycle, not only in transitional and coastal environments but also far offshore in the open ocean. Our results indicate that during diverse periods of ice sheet growth and decay, including large-scale glacial retreat and times of glacial instability within warmer climate intervals, reactive Fe can be transported beyond just coastal and shelf regions. Of note, Fe delivery does not occur during the Holocene at any of our sites, suggesting that retreat of tidewater glaciers away from the shelf shuts off the reactive Fe delivery pathway. While Svalbard glaciers have generally retreated to the extent that a future perturbation to the Fe cycle would not be expected, both Greenland and Antarctica ice retreat are potentially important pathways of Fe delivery to the Fe-limited Southern Ocean and northern North Atlantic. Our results indicate that systems are most sensitive during the retreat and expansion of glacial systems, and rapid retreat of marine terminating glaciers is currently occurring^78,79^, which may result in significant biogeochemical perturbations.

## Methods

Samples were retrieved on the RV Polarstern during the Transitions in the Seasonal Sea Ice Zone (TRANSSIZ) expedition (PS92) in spring 2015^80^ (Fig. 1). The present study focuses on the 8.6 m kastenlot core (PS92/39-2), recovered from 1464 m water depth at 81.94983 ^o^N 13.82833 ^o^E. Additional results are shown from cores PS92/45-2 and PS92/0054-1. The study focuses on the upper 6 m of this core, which covers the interval since the last interglacial. Scanning XRF data was produced using an Avaatech XRF core scanner, which uses Rh-radiation that moves through a He-flushed chamber. The sediment surface was covered with a 4 μm thick Ultralene foil for analysis. The down-core slit size was 10 mm, and the cross-core slit size was 12 mm with a 10 mm step. The 10 kV analyses were made with no filter and a 10 s measuring time. Color images were acquired with a Jai-L-107CC 3 CCD RGB Line Scan camera installed on the XRF core scanner. Cores were sampled at sea every 5 to 10 cm, frozen at −20 ˚°C, and subsequently freeze-dried. Samples were then powdered in an agate mortar and pestle. For all analyses, 10% of samples were analyzed in triplicate. Sediment samples were fused into borate glass beads and were analyzed for total elemental contents, including Fe, Mn, P, and Al, using quantitative X-ray fluorescence (XRF) with a Philips PW-2400 WD-XRF spectrometer calibrated with 53 geostandards at the University of Oldenburg^81^. Replicate analyses reproduced with average errors of <3%. Barium excess concentrations were calculated as follows:

1 $${{{\rm{Ba}}}}_{{{\rm{Ex}}}}={{{\rm{Ba}}}}_{{{\rm{sample}}}}{{{-}}}{{{\rm{Al}}}}_{{{\rm{sample}}}}*({{{\rm{Ba}}}}_{{{\rm{avg}}}.\; {{\rm{shale}}}}/{{{\rm{Al}}}}_{{{\rm{avg}}}.\; {{\rm{shale}}}})$$

A sequential Fe extraction methodology was adapted from Poulton and Canfield (2005) to determine the quantity of reactive Fe phases. Acid-soluble Fe was extracted by 1 M HCl for 0.5 h. Crystalline Fe (oxyhydr)oxides were extracted by citrate-buffered Na-dithionite (pH 4.8) for 2 h, and magnetite Fe was extracted by ammonium oxalate (pH 3.2) for 6 h. Additionally, poorly crystalline Fe oxyhydroxides (ferrihydrite) were extracted from a separate sample split using a 24-h ascorbic acid solution buffered at pH 7.5^49^. Iron contents were analyzed by flame atomic absorption spectroscopy at the University of Leeds. Average errors on individual measurements were <5%.

Sequential P extractions were adapted from the SEDEX protocol (Ruttenburg et al., 1992). In short, loosely-sorbed P was extracted using MgCl_2_ (pH 8; 8 h). Iron-bound P was then extracted with citrate dithionite bicarbonate (pH 8; 8 h). Detrital and authigenic P were extracted with 1 M sodium acetate (pH 4; 6 h) and 10% HCl (16 h), respectively. A residual P phase is determined as the total P content minus measured phases and includes organic P. Extracted phases were analyzed for P on a spectrophotometer via the phosphomolybdate blue method, with the solutions adjusted to a pH of 1–2 where necessary, with the exception of the dithionite solution, which was analyzed on a Thermo Fisher iCAP 7400 Radial ICP-OES, due to interference between the solution matrix and the phosphomolybdate complex.

Fe isotopes were measured on a total sediment digestion. Samples were ashed at 550 °C and then dissolved in HNO_3_-HF-HClO_4_, followed by evaporation to dryness. Boric acid was then added, and samples were subsequently evaporated to dryness again. The sample was then redissolved in HNO_3_ on a hot plate. For analyses of Fe isotope ratios, Fe was separated using 1.8 mL AG1X8 anion resin (100–200 mesh, Bio-Rad) according to the protocol of Dauphas et al. (2009)^82^. All acids used were of ultrapure quality (Fisher Optima or distilled). Iron isotope ratios were determined using the high-resolution multi-collector ICP-MS (Thermo-Scientific Neptune *Plus*) at ICBM^83^. A Ni isotope standard solution (NIST 986) was used for instrumental mass bias correction^82,84^. Analyses followed the standard-sample-standard bracketing method. The results are given in delta notation:

2 $${{{\rm{\delta }}}}^{56}{{\rm{Fe}}}({{ \textperthousand }})=[({\scriptstyle{{56}}\atop} \!{{\rm{Fe}}}/{\scriptstyle{{54}}\atop} \!{{{\rm{Fe}}}}_{{{\rm{sample}}}})/({\scriptstyle{{56}}\atop} \!{{\rm{Fe}}}/{\scriptstyle{{54}}\atop} \!{{{\rm{Fe}}}}_{{{\rm{IRMM}}}-524})]\, {{\bullet }}\, {10}^{3}$$

relative to the international Fe isotope standard IRMM-524 that was analyzed before and after each sample. The reference materials AC-E (granite, IWG-GIT), SDO-1 and SGR-1 (both shales, US Geological Survey) were used to determine the accuracy of the Fe separation and measurement procedures. Measured values for AC-E (δ^56^Fe = 0.349 ± 0.023 ‰ 2 SD, *n* = 9), SDO-1 (δ^56^Fe = 0.020 ± 0.026 ‰ 2 SD, *n* = 5) and SGR-1 (δ^56^Fe = 0.113 ± 0.028 ‰ 2 SD, *n* = 3) interspersed between the samples were in agreement with recommended values of 0.322 ± 0.036 ‰ for AC-E^82^, 0.023 ± 0.028 ‰ for SDO-1^68^ and 0.15 ± 0.06 ‰ for SGR-1^85^. The procedural Fe blanks were insignificant (i.e., <0.1% of the sample with the lowest Fe content). See Böning et al. (2020) for more details on column chemistry and ICP-MS measurement conditions.

Mössbauer spectra were collected on four samples, including a high and low Fe content sample from the upper meter and deeper within the record. Powder samples were analyzed at both room temperature (295 K) and at 4 K, using a MS4 Mössbauer spectrometer (SEE Co., Edina, MN) in conjunction with a closed-cycle cryostat (SHI-850, Janis Research Co., Wilmington, MA). Spectra were collected in transmission geometry applying a constant acceleration mode of the Co-57 source (Ritverc) and calibrated against a 0.7 µm α-Fe(0) foil. Spectral analysis was carried out with the Voigt-based fitting routine of the software package Recoil.

X-ray diffraction pattern analyses have been done in the laboratories of the research group Crystallography & Geomaterials Research (University of Bremen, Dept. of Geosciences). Dried bulk samples were grounded to a fine powder ( < 20 µm particle size) and prepared with the Philips backloading system. A thorough preparation commonly increases reproducibility of the results; however, the standard deviation given of ±5% can be considered as a general guideline for mineral groups with >20% clay fraction^86^. In addition, the determination of well-crystallized minerals like quartz, calcite or aragonite can be done with better standard deviations (±1–3%)^87^. The X-ray diffractograms have been measured on a Bruker D8 Discover diffractometer equipped with a Cu-tube (k_α_ 1.541, 40 kV, 40 mA), a fixed divergence slit of ¼°, a 90 sample changer and a monochromatisation via energy discrimination on the highest resolution Linxeye detector system. The measurements were done as a continuous scan from 3–65° 2θ, with a calculated step size of 0.016° 2θ. Mineral identification has been done by means of the Philips software X’Pert HighScore™ software X’Pert HighScore™ Vs. 1.2^88^. This was followed by full quantification of the mineral assemblage of the bulk fraction via the QUAX full pattern method^87^.

## Supplementary information

Supplementary Information

Peer Review file

## Data Availability

Data presented in this paper are available on Zenodo (10.5281/zenodo.20101890).
