# Supplementary material for: Svalbard-barents ice sheet instability enhanced delivery of reactive iron to the ocean
Source: Nat Commun. 2026 Jul 1;17:8164. doi: 10.1038/s41467-026-75133-2 (PMC13458693; doi:10.1038/s41467-026-75133-2)
Supplement: Supplementary file 1 [file 41467_2026_75133_MOESM1_ESM.pdf]

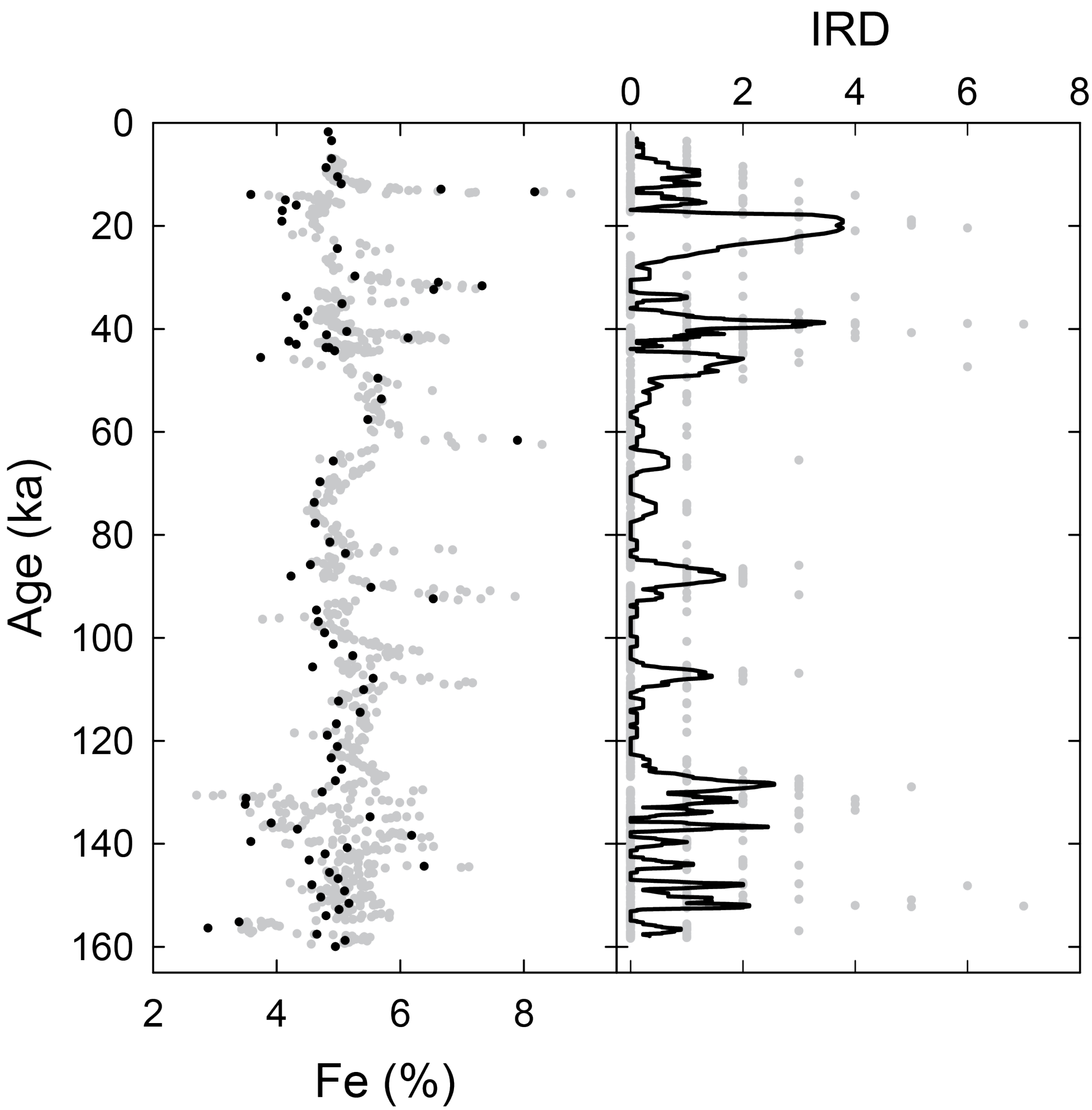

Supplementary Fig. S1: Fe concentrations (wt. %) and ice rafted debris (IRD) counts with a 5 pt running smooth from PS92/39-2.

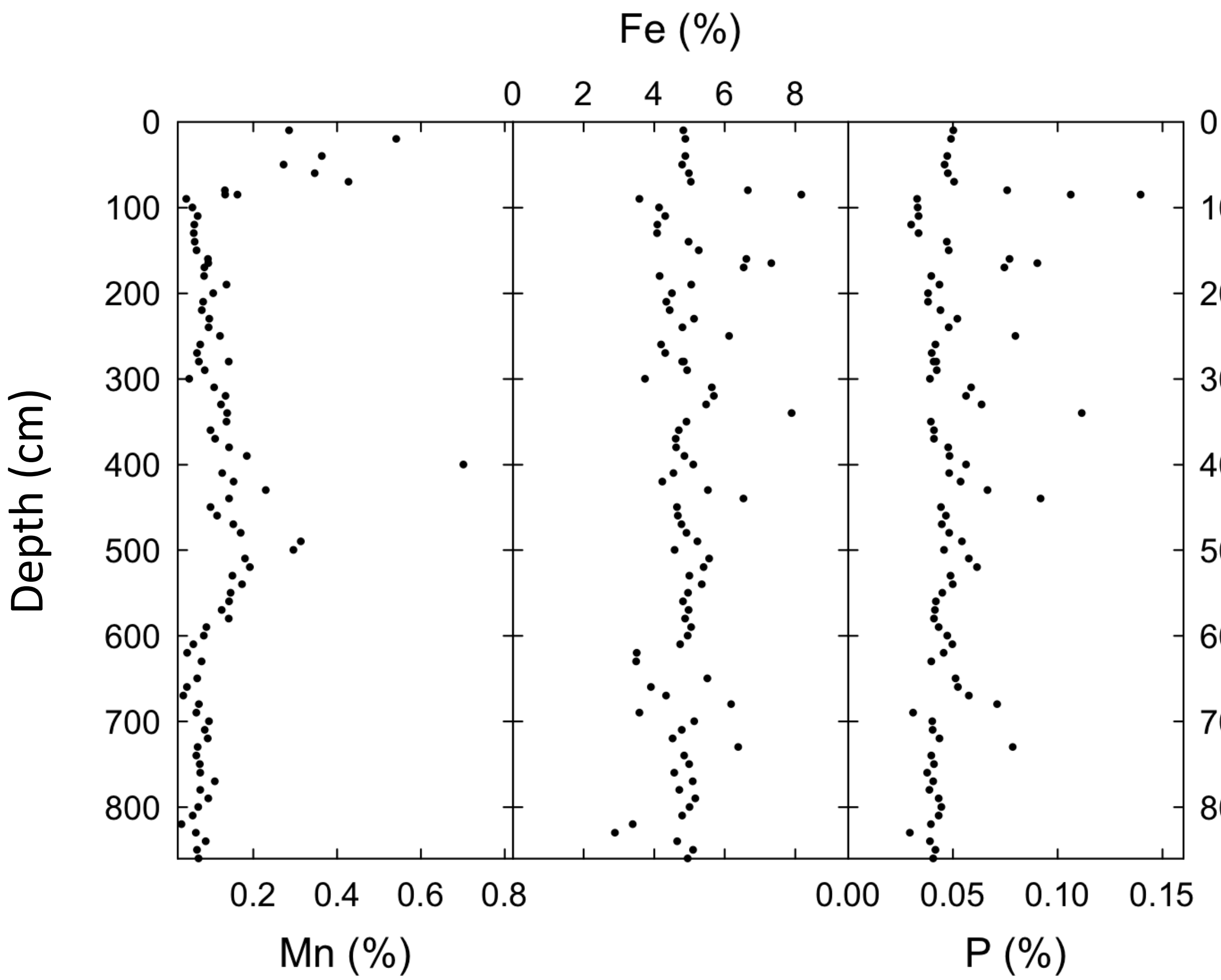

Supplementary Fig. S2: Total Mn, Fe, and P concentrations for PS92/39-2 from XRF analysis. Manganese concentrations do not correlate strongly with Fe concentrations. In particular, Mn is largely enriched within the Holocene sediments and Mn is not enriched within laminated, Fe- and P-rich layers.

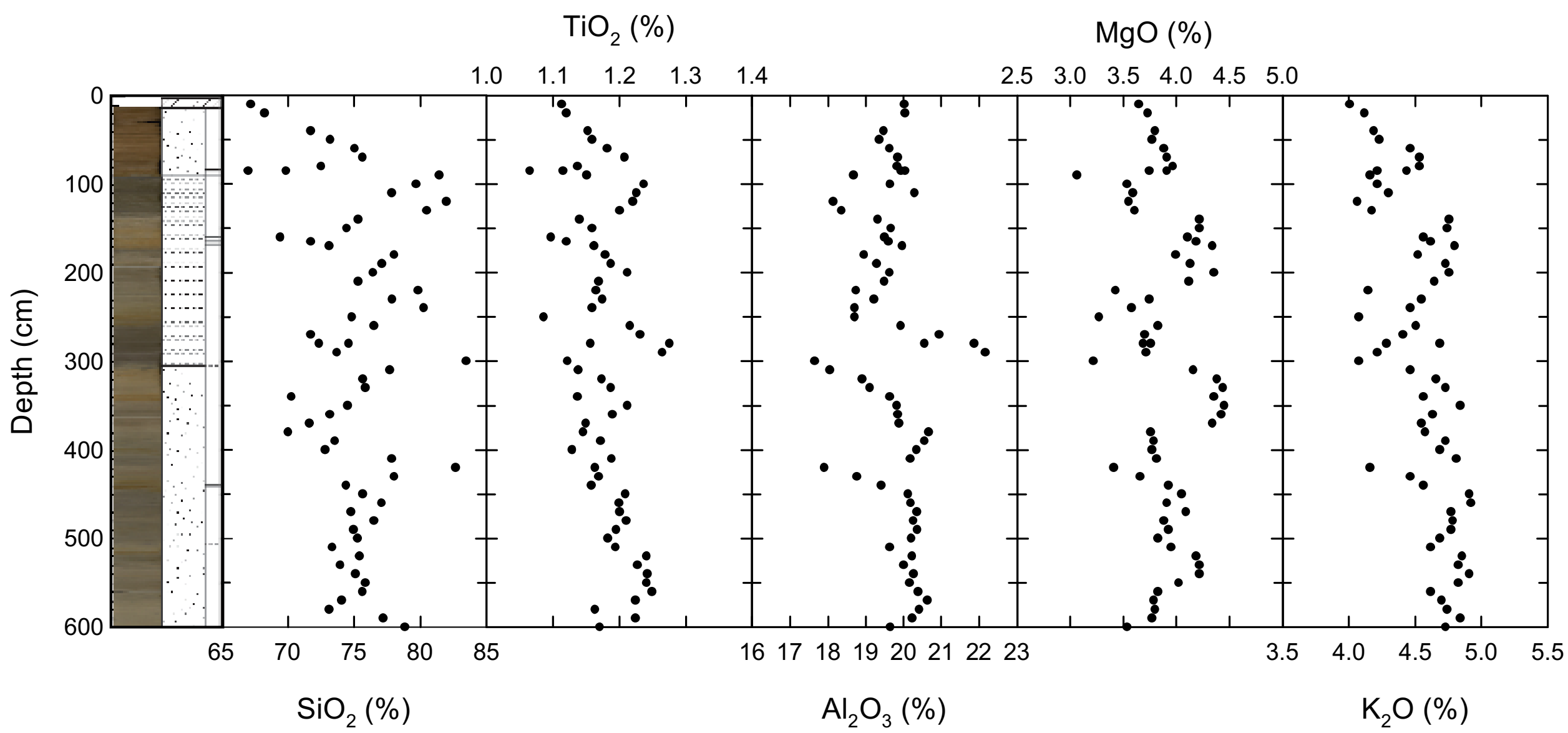

Supplementary Fig. S3: Lithogenic elements PS92/39-2 normalized to total concentration of lithogenic elements to control for concentration changes forced by changes in Fe, Mn, P, TOC, CaCO3. This plot highlights the relative change in each element and the fact that the Fe-rich layers are still distinct when dilution by authigenic and biogenic phases is considered.

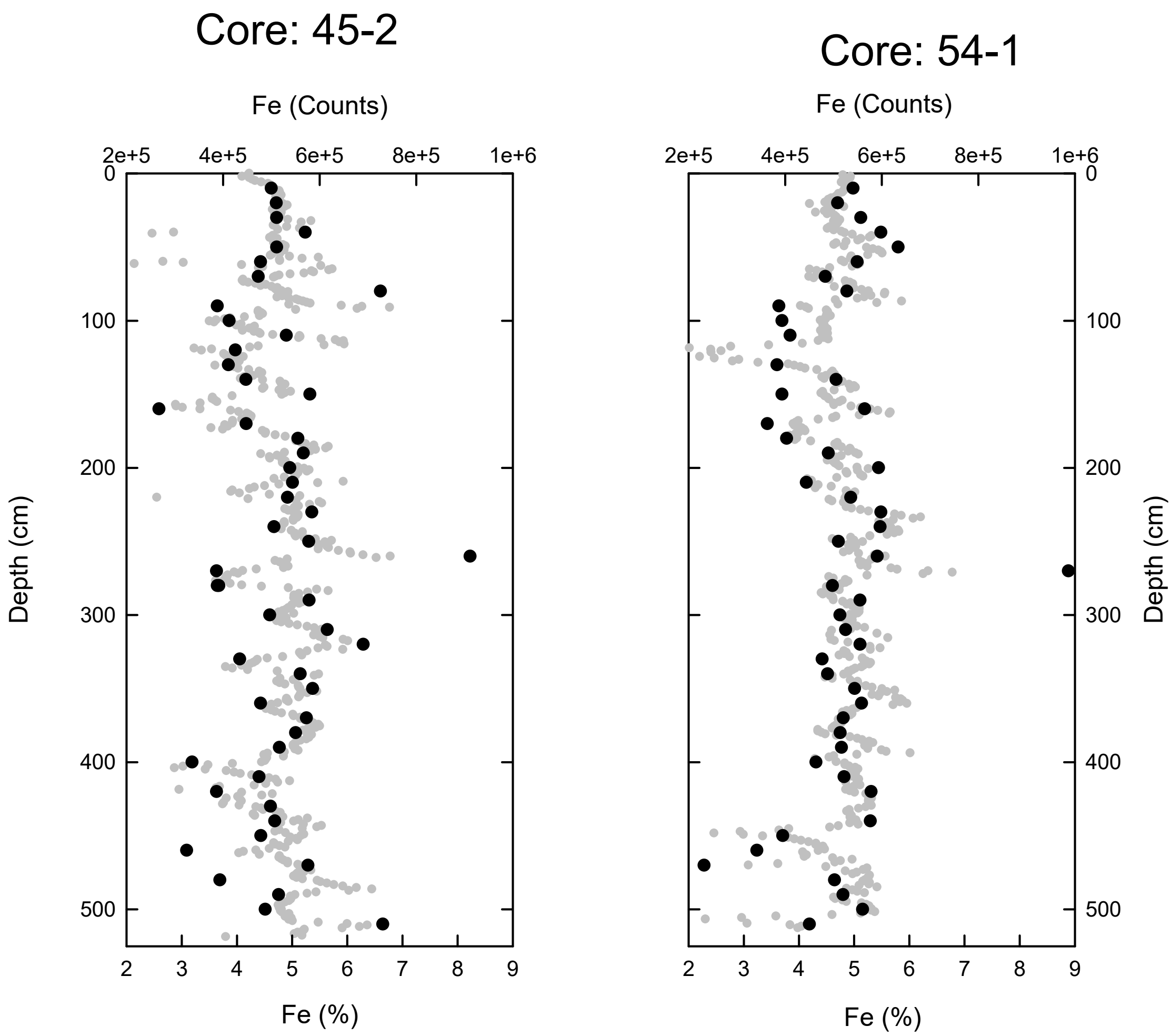

Supplementary Fig. S4: Scanning XRF and quantitative XRF data from PS92/45-2 and PS92/54-1 illustrating that Fe-rich layers similar to those found in PS92/39-2 are common across the region. These layers have a similar total Fe total (> 8wt.%) as the layers within PS92/39-2.
