# Supplementary material for: Svalbard-barents ice sheet instability enhanced delivery of reactive iron to the ocean
Source: Nat Commun. 2026 Jul 1;17:8164. doi: 10.1038/s41467-026-75133-2 (PMC13458693; doi:10.1038/s41467-026-75133-2)
Supplement: Supplementary file 2 [file 41467_2026_75133_MOESM2_ESM.pdf]

nature portfolio

Peer Review File

Svalbard-Barents Ice Sheet instability enhanced delivery of reactive iron to the ocean

Corresponding Author: Dr Allyson Tessin

This file contains all reviewer reports in order by version, followed by all author rebuttals in order by version.

Version 0:

Reviewer comments:

Reviewer #1

(Remarks to the Author)
Summary:
Glacial meltwater has been observed to have high concentrations of iron in subglacial outflows, derived from rock-water weathering beneath warm-based glaciers. However, the role of glaciers in the iron cycling is still unclear due to a paucity of data and because much of the glacially-derived iron is trapped within estuaries/fjords before delivery to the open ocean where it might impact marine carbon cycling as a micronutrient for phytoplankton and means of carbon burial through mineral association. Tessin et al. use iron concentrations in a dated core from the Yermak Plateau to explore the input of reactive iron from the Svalbard-Barents Ice Sheet during periods of ice sheet instability. The authors found that during periods of ice sheet instability large inputs of reactive iron into the sediments occurred. This indicates that a) glaciers may be a important source of reactive iron to the oceans during ice advance and retreat, and b) that glacially-derived iron may be delivered far offshore (100s of km), potentially impacting iron-limited or iron-poor marine ecosystems. The work has implications for contemporary ice sheet iron delivery given the potentially rapid wastage of the Greenland and Antarctic ice sheets in current climate projections.

General comments:
I read this manuscript with great interest. The hypothesis presented, that ice sheets could be large sources of iron to coastal systems, is of great interest, and could have large implications for how we understand the cryosphere in global biogeochemical cycles both now and over the Quaternary. The data is of high quality and is fascinating. The arguments made are also compelling as written (with the caveats below) and I love the ambition of the manuscript. I am therefore mostly going to play devil's advocate in the hope of tightening the main arguments presented. Could the authors make a better case for upscaling these findings from one core? And is there any evidence from contemporary environments that reactive iron concentrations can reach such high values? For example, I do not know of any glacial environments sampled where ascorbate iron can reach over 1% by weight. Interestingly, the red sandstones invoked as being a possible source of high Fe do not typically have very high ascorbate extractable Fe concentrations (see (Hopwood et al., 2017)). The authors present several ways in which enrichment might work, but may want to provide additional information on how generation and long-term preservation of such high ascorbate iron concentrations can occur, particularly as we would expect aging of ascorbate iron to less reactive, more crystalline forms (such as those extracted by dithionite) over time (as the authors point out). One source of confusion for a reader could be the use of two sets of Fe extractions (ascorbate vs the others) and it's not currently clear in the manuscript how these are similar or differ, and what they both or individually might be extracting. Finally, would it be possible to do any back-of-the-envelope calculations for what the flux of reactive iron to the ocean during some of these pulses might be (e.g. using deposition rate and Fe concentration)? Even if crude or localized, this could provide some valuable information on the magnitude of iron delivery during ice sheet instability in ice distal regions. I recommend publication after some minor to moderate corrections.

Specific comments:
L48: The correct term here would be "subglacial meltwater outflow" or just "meltwater" rather than subglacial melt, because much of the water is derived from the surface melting, not subglacial melt. Probably (Hawkings et al., 2014) would be a more useful citation here than Statham et al., as it includes comparable particulate Fe data to that presented in this study, while Statham only present dissolved data (that aren't actually that high).
L50-51: Similar to above, these are good references, but neither explicitly address, or measure reactive Fe phases. I would stick to references that more directly present this evidence.

L52: I think better to say “pass through estuaries/fjords before entering the shelf and open ocean”. In my mind, these environments are already the coastal ocean.
L59: Would “nitrogen” be more accurate here than nitrate?
Figure 2: I could see the panel lettering here. Acronyms need to be explained in the caption.
L100-101: What do you mean here? And a reference would be appropriate.
Figure 3: Need uncertainty bars on isotope ratio measurements.
L125-127: Or, to play devil’s advocate, that Mn is more mobile and does not precipitate at the same depth as Fe in the core making that interpretation not quite so simple?
L155-156: Poorly ordered and amorphous, but not nano-goethite. Perhaps that’s not the point being made, but it feels like that this sentence is meant to relate to the previous.
L174-180: Small differences in d56Fe measurements on sediment are always quite difficult to interpret, particularly as uncertainty hasn’t been presented here. Are you convinced these differences are significant? What is the fractionation you would expect from dissolution and reprecipitation? For example, Fe reduction is invoked for lower d56Fe values, but this is usually in the dissolved phase. Presumably you are arguing that this lighter Fe reprecipitates to for the Fe-rich layers? To be clear, I’m not saying you are wrong with the interpretation here, but I’m not sure it adds a huge amount to the data interpretation.
L213: recommend “due to flocculation and sediment at low salinities”
L218-220: As above, I’m not sure I would overrely on the Fe isotope data for this kind of interpretation (which takes a bit of a leap of faith). The differences are relatively small, and there could be other influences such as bedrock, or processing in subglacial systems.
L220-222: How much of the shelf would be exposed at these points in time? And would glaciers discharge into fjords first, or directly off the shelf? In either case this interpretation of benthic iron shuttling might need some more thought or clarification in the text.
L262-264: This might also need some additional thought. There are still many tidewater glaciers in Svalbard, so this doesn’t quite explain it all. Is it more the emergence of fjords as sediment traps or elemental sinks? I.e. the glaciers need to terminate outside of fjord systems onto the shelf or (in some instances) off the shelf.
L293: More details needed here. What type of AAS? Precision/accuracy?
L299: How was P measured?
L300-301: Is the HCl extraction data included in the manuscript?

References cited:
Hawkings, J.R., Wadham, J.L., Tranter, M., Raiswell, R., Benning, L.G., Statham, P.J., Tedstone, A., Nienow, P., Lee, K., Telling, J., 2014. Ice sheets as a significant source of highly reactive nanoparticulate iron to the oceans. Nat. Commun. 5. https://doi.org/10.1038/ncomms4929
Hopwood, M.J., Cantoni, C., Clarke, J.S., Cozzi, S., Achterberg, E.P., 2017. The heterogeneous nature of Fe delivery from melting icebergs. Geochemical Perspectives Letters 3, 200–209. https://doi.org/10.7185/geochemlet.1723

Reviewer #2

(Remarks to the Author)
Overall comment
The paper presents nice, original, and compelling data, demonstrating significant pulses of iron-rich material that correlate with major deglaciation and/or periods of instability. These results are the novelty of this paper, as it supports the ongoing hypothesis that during ice sheet instability or deglacial periods, large quantities of reactive glacial iron are exported to the marine system, with implications for marine productivity and carbon export. This is a 'hot' topic currently, and the implications of which are of interest to scientists in several fields. The authors present important supporting data, and interpret the layers of reactive Fe as bioavailable.

My main concerns are
- Although the authors present a nice dataset, the paper narrative needs work to be as accessible and clear/consistent as possible. There are several grammatical errors and sentences in need of rephrasing. The paper would benefit from some editing (some text is not needed) to be more precise and concise, tackling only what is most relevant to the central hypotheses of the paper
- There is a lack of error propagation and reproducibility in results that is not in line with the journal requirements. Particularly, Fe sediment extractions are notoriously accompanied by relatively high uncertainty, and so authors must state the reproducibility of results from each technique for this material to be of publishable standard.
- The paper discusses the reactivity of deposited sediments, and connects reactivity with bioavailability. However, there are several significant assumptions made here that are not clearly addressed, such as the post-depositional changes to Fe reactivity, the link between reactivity and bioavailability which is not tested, and the lack of general evidence that some ‘reactive’ Fe is actually bioavailable e.g. nano-goethite. The broader body of evidence that certain nanoparticles (Fe(II)-rich glaciogenic sediments, ferrihydrite) are bioavailable also remains slim (albeit growing) and the authors don’t reference all the key papers! This hypothesis has not, to my knowledge, been tested on deep marine sediments. I recommend that the authors need to carefully define what they mean by ‘reactive’ and ‘bioavailable’, and discuss the post-depositional diagenesis that may have occurred
- The two methods – Fe sediment leaches and Mossbauer spectroscopy - used to determine iron phase speciation and reactivity - do not give the same results, but the evaluation of this feels incomplete.

- I’m not overly convinced the Fe isotope dataset adds much, and the authors also describe in the methods a data subset of

Fe isotopes (acid-leached pool) that is not presented in the results/figures

Abstract – haven't defined Fe as iron. Either keep as 'iron' in abstract and define in intro, or define Fe on line 21.
Line 30 – I think this claim has too many assumptions. It suggests that all reactive Fe in sediments is bioavailable. Only certain reactive Fe forms have been shown to be somewhat available to phytoplankton, and this hasn't been tested on these deep marine sediments (or similar elsewhere). Additionally there is the matter of post-deposition changes to reactivity. I suggest that you adjust your language to say that some of the iron deposited may have been somewhat bioavailable at the time of deposition, and be careful throughout the paper in this interpretation since the sediment bioavailability has not been tested.
Line 47 – iceberg is one word
Line 55 – not sure if this is called into question as even if loss 80 – 98 % of dissolved Fe, the remaining can still be biologically important, and plenty of papers show that meltwater delivers biologically important dFe (and particulate Fe) to the surface ocean. So despite the salinity gradient, Fe delivery to the marine system from glacial sources is shown to prevail. Several other studies link benthic supply of Fe from high latitude shelf sediments to increased primary productivity. Also, this paper discusses particulate Fe really, so what about this? Particularly since you suggest certain particulate Fe is bioavailable.
Line 57 – 61 needs work. By southern high-latitude oceans – do you mean the Southern Ocean? Large regions of the Southern Ocean are critically light limited in winter as well, whilst large regions of the Arctic are nitrate limited during summer. You need a reference for Southern Ocean iron limitation. Browning and Moore (2023) Nat Comms, and Moore et al (2013) Nature look at global nutrient limitation so both of these would be appropriate to show patterns of nutrient limitation in both polar regions. Generally, are you talking about present day or over glacial/interglacials as this is not clear? The focus of the paper is the last 135ka so make it clear to the reader.
Line 63 – 'nitrate-limited regions are largely controlled by iron supply' – this doesn't make sense. You mean the productivity in these regions is controlled by iron supply?
Line 66 – would be nice to have some melt/retreat rates included here.
Line 73 – 'biogeochemically reactive Fe'. This is the only time you use this phrase and there is no explanation of what is meant by this. I think this is really important to define what you mean when you say reactive early on, since the audience is quite broad. Are you talking about material that readily chemically dissolves in seawater, or material that is bioavailable? These are two distinct processes and are important for your focus on particulate/dissolved Fe and the link to the sediments.
Line 85 – starting the results by describing the age model feels clunky. To a broad audience this isn't that clear either – perhaps most of this could be moved to the methods? Iron is the central tenet to this paper so put this up front.
Line 92 – perhaps put 'see methods' rather than listing undefined extraction methods that are probably unknown to much of a broad audience.
Line 93 – can you put this in context? As that's a really high reactive Fe content but that's not clear here.
Line 98 – quantitatively define 'elevated' FeT contents relative to your background
Line 100 – 'based on modelling of Mossbauer results', 'ordered fe-oxyhydroxide… are reactive'. This is too vague. I think again you need to explain the implications of a reactive Fe pool and what's considered biologically important if that's what you're invoking.
Line 103 – this isotope value is a little abrupt. Has this been previously defined for reader.
Line 105 – 'P(T) tracks total Fe(T)' – this is too vague. Is there a statistically significant correlation?
Line 126 – comma after front.
Line 132 – comma after diverse
Line 136 – 'delivers pink sediments' – what does this mean?
Line 140 – 'the presence of tidewater…' this is vague and you don't discuss the implications of this.
Line 146 – Yoshida et al. don't include goethite in their culturing experiments. Goethite is a more crystalline Fe (oxyhydr)oxide than the feed stock they use. They age pure ferric iron stock in seawater over 1 day to 3 weeks – defining this as amorphous Fe (oxhydr)oxides. I think there's some confusion here between these definitions, and to my knowledge there has been no culturing study that shows goethite as bioavailable. See also Wyatt et al (2024) PNAS for culturing experiments with glaciogenic material, and Jones et al. (2025) Nature Communications for determining particulate Fe speciation delivered by glacial meltwaters.
Line 164-165 – do you mean 370 cm?
Line 169 – this sentence and the following don't make sense and have grammatical errors.
Line 174 – I am not an Fe isotope expert but this analysis feels a little incomplete.
L:ine 186 – comma after water column. Periodic input of increased reactive Fe? May act to trap P, rather than prevented the flux of P, perhaps. There may be interaction and redissolution of these minerals back into the water column, so is 'prevented the flux of P' totally accurate?
Line 190 – glaciers are key generators of amorphous Si and glacial Si export is dominated by amorphous silica, so these peaks could also be interpreted as spikes in glacial Si delivery e.g. Hatton et al., 2019 (Proceedings of the Royal Society A).
Line 205 – Studies linking particulate Fe delivery by meltwater are relevant… Suggest Jones et al. (2025) Nature Communications, in which they directly measure particulate Fe and Fe speciation, finding a prevalence of Fe(II) and mixed valence Fe delivery by glacial meltwater to the surface ocean.
Line 210 – unclear phrase. Has Fe mineral deposition been proposed as a proxy, or meltwater?
Line 260 – 'delivery of reactive Fe significantly offshore' – what does this mean? That the Fe travels a large distance? I don't think this is the right wording.
Line 263 – 'Fe delivery does not occur during the Holocene' – Don't you mean that Fe delivery is not recorded in the records at your site?
Line 267 – the final two references feel a bit out of place / poorly ordered. Suggest incorporating with the previous statement.

Methods
Line 287 – were sediments freeze dried? This has been shown to change the mineralogy and reactivity of iron (oxhydr)oxides (Raiswell et al. 2010). No details of replicates or reproducibility / error propagation are provided.
Line 300 – what was the reproducibility of isotope measurements? Did you run any replicates? This needs stating and the error bars provided in Figure 3. Do you present the 0.5 M 1 hr HCl isotopes as I don't see them in your main or supplementary figures or results, but it would be helpful to have these data. Also, why is this sediment extraction protocol different to the Fe reactive phase 1M 0.5 hour extraction? This therefore represents a different Fe pool.

Line 322 – can you explain why the Mossbauer data was not accompanied by standards? Considering the discrepancy between this and the Fe extractions, some discussion of error propagation of both of these methods is warranted. Does the Voigt-based fitting routine use built in standards for spectral analysis or were standards ran by you?
Line 329 – as above, for XRD. Did you measure any standards to verify?

Figures/tables
Figure 3 – Add error bars/comment on errors in caption, for 3a and 3c.

Version 1:

Reviewer comments:

Reviewer #1

(Remarks to the Author)
I thank the authors for working hard on this manuscript, which I think is much improved. I'd be very happy to see this published with no further edits needed. This will be a valuable study in the literature.

Reviewer #2

(Remarks to the Author)
The authors have done a great job of revising the manuscript and I have no further comments or revisions.

Open Access This Peer Review File is licensed under a Creative Commons Attribution 4.0 International License, which permits use, sharing, adaptation, distribution and reproduction in any medium or format, as long as you give appropriate credit to the original author(s) and the source, provide a link to the Creative Commons license, and indicate if changes were made.
In cases where reviewers are anonymous, credit should be given to 'Anonymous Referee' and the source.
The images or other third party material in this Peer Review File are included in the article's Creative Commons license, unless indicated otherwise in a credit line to the material. If material is not included in the article's Creative Commons license and your intended use is not permitted by statutory regulation or exceeds the permitted use, you will need to obtain permission directly from the copyright holder.
To view a copy of this license, visit https://creativecommons.org/licenses/by/4.0/

Reviewer 1
General comments:

Could the authors make a better case for upscaling these findings from one core?

We agree that this is a weakness of the paper. To address this concern, we have added results from two other gravity cores in the region (45-2 and 54-1) in the supplemental material that were collected on the same expedition. All three cores contain evidence for layers of Fe-rich sediment deposition. This addition is reflected in an updated map, new supplemental data, and the methods section, as well as within the discussion.

And is there any evidence from contemporary environments that reactive iron concentrations can reach such high values? For example, I do not know of any glacial environments sampled where ascorbate iron can reach over 1% by weight. Interestingly, the red sandstones invoked as being a possible source of high Fe do not typically have very high ascorbate extractable Fe concentrations (see (Hopwood et al., 2017)).

Enrichments of ascorbate extractable-Fe in fjord sediments have been observed within this range (0.98 wt. %; Laufer et al., 2020). We have added a statement about the enrichments relative to bedrock and glacial source material and how this indicates that either an enrichment mechanism is required, or remobilization of fjord or shelf Fe is a likely path, with new text reading: "These Fe_{asc} contents exceed Fe_{asc} found within most glacial source materials on Svalbard and the Devonian bedrock (Hopwood et al., 2017; Laufer-Meiser et al., 2021), which demonstrates the need for a mechanism that increased Fe reactivity between the glacial source and the Yermak Plateau. Similarly high Fe_{asc} contents (up to 1 wt%) have been observed in fjord sediments (Laufer et al., 2020), which suggests that remobilization of fjord or shelf sediments could result in Fe_{asc}-rich sediment deposition on the plateau."

The authors present several ways in which enrichment might work, but may want to provide additional information on how generation and long-term preservation of such high ascorbate iron concentrations can occur, particularly as we would expect aging of ascorbate iron to less reactive, more crystalline forms (such as those extracted by dithionite) over time (as the authors point out).

We agree and details have been added about how this amount of Fe_{asc} compares to other systems, as well as clarifying that the aging of ferrihydrite to goethite appears to be inhibited

in sediments, potentially due to phosphate or organic carbon complexation, have been added to the discussion of the ascorbate text.

One source of confusion for a reader could be the use of two sets of Fe extractions (ascorbate vs the others) and it’s not currently clear in the manuscript how these are similar or differ, and what they both or individually might be extracting.

We agree that this could be confusing. Within both the methods and then discussion, we have attempted to clarify the difference.

Finally, would it be possible to do any back-of-the-envelope calculations for what the flux of reactive iron to the ocean during some of these pulses might be (e.g. using deposition rate and Fe concentration)? Even if crude or localized, this could provide some valuable information on the magnitude of iron delivery during ice sheet instability in ice distal regions.

We agree in the value of this calculation. The following text has been added:

Layers of Fe-rich sediments of between 8-10 wt.% Fe are found within three cores across the Yermak Plateau, despite different water depths and oceanographic settings, supporting that these represent intervals of changing sediment dynamics across the region. These layers are an average of 3 cm thick and represent an increase of 4 wt.% Fe over the baseline Fe content. If we assume that the area between our three sites (~7000 km^3) experiences increased Fe delivery, this represents 13.4 Tg of excess particulate Fe input, which is approximately ~10 years of the average particulate Fe modern input from Greenland or >100 years of the average modern input from Antarctica.

Specific comments:
L48: The correct term here would be “subglacial meltwater outflow” or just “meltwater” rather than subglacial melt, because much of the water is derived from the surface melting, not subglacial melt. Probably (Hawkings et al., 2014) would be a more useful citation here than Statham et al., as it includes comparable particulate Fe data to that presented in this study, while Statham only present dissolved data (that aren't actually that high).

Both of these changes were made.

L50-51: Similar to above, these are good references, but neither explicitly address, or measure reactive Fe phases. I would stick to references that more directly present this evidence.

This text was corrected as suggested.

L52: I think better to say "pass through estuaries/fjords before entering the shelf and open ocean". In my mind, these environments are already the coastal ocean.

We agree and this text was updated.

L59: Would "nitrogen" be more accurate here than nitrate?

These references do refer to nitrate limitations.

Figure 2: I could see the panel lettering here. Acronyms need to be explained in the caption.

The figure caption has been updated.

L100-101: What do you mean here? And a reference would be appropriate.

Here, we refer to (1) the portion of Fe that ordered magnetically only at 4 K (labelled LHT-OxHy in Figure S4) and with Mössbauer parameters (Table S1) consistent with Fe (oxyhydr)oxides such as goethite or ferrihydrite (reference: Thompson et al, 2025). Since ordering occurred below room temperature, any goethite comprised in this component is poorly ordered and/or of small size, in analogy to the interpretation applied by Thompson et al, 2011. Hence, we classify this portion as 'reactive'. (2) The unordered Fe(II)/Fe(III) minerals are the portion of Fe that appears as a collapsed feature in the Mössbauer spectra collected at 4 K (labelled LHT-collapsed in Figure S4). As we argue in the supplemental information, we assigned this component to disordered and/or short range ordered Fe phases, which we, again, classify as 'reactive'.

To clarify these assignments and the summary statement, we updated the text on the Mössbauer spectroscopic results in the Results section (lines):

Based on Mössbauer spectroscopy, Fe (oxyhydr)oxides increase in samples with elevated FeT contents (51-62% of the Fe pool, as compared to 4-23%; yellow bars in Figure 3). This component exhibits Mössbauer parameters (Table S1) consistent with

ferrihydrite and/or goethite, with the latter being poorly ordered and/or of small size as indicated by its magnetic ordering occurring well below room temperature (reference: Thompson et al, 2025). We consider these poorly ordered Fe (oxyhydr)oxide(s), together with the incompletely ordered component we observed at 4 K (white bars in Figure 3), as reactive Fe phases.

Figure 3: Need uncertainty bars on isotope ratio measurements.

Error bars have been added to Figure 3.

L125-127: Or, to play devil's advocate, that Mn is more mobile and does not precipitate at the same depth as Fe in the core making that interpretation not quite so simple?

We agree and softened the language to just focus on the observation.

L155-156: Poorly ordered and amorphous, but not nano-goethite. Perhaps that's not the point being made, but it feels like that this sentence is meant to relate to the previous.

We clarified the text to specify that the ascorbate is assumed to mainly target 2-line ferrihydrite and acknowledged which evidence supports the presence of ferrihydrite vs. goethite.

L174-180: Small differences in d56Fe measurements on sediment are always quite difficult to interpret, particularly as uncertainty hasn't been presented here. Are you convinced these differences are significant? What is the fractionation you would expect from dissolution and reprecipitation? For example, Fe reduction is invoked for lower d56Fe values, but this is usually in the dissolved phase. Presumably you are arguing that this lighter Fe reprecipitates to for the Fe-rich layers? To be clear, I'm not saying you are wrong with the interpretation here, but I'm not sure it adds a huge amount to the data interpretation.

We agree that the isotope signal is small and we do not intend to overinterpret it. We have added text to the manuscript acknowledging the small signal and error bars within the figure. We have also updated the text and isotope figure to be clearer about the proposed process and how it has been interpreted within Arctic sediments previously. We also aimed to be clear that it is a piece of evidence that may support our proposed model of remobilization of Fe-rich material from more proximal environments. New text is as follows: "Fe_T isotope results (Figure 3) support that there was an intermediate step of deposition within fjord and

shelf sediments followed by remobilization to coastal waters and beyond. Dissimilatory Fe reduction occurs in organic-rich Arctic shelf sediments resulting in non-lithogenic Fe (oxyhydr)oxide enrichments at the sediment surface (e.g. Strekopytov, 2003). Lower δ^{56}Fe values observed further into the Arctic basins has been interpreted as a basin-ward transport of these shelf sediments (Meinhardt et al., 2016). Although the range of isotope values is small, Fe_T-rich sediment layers on the Yermak Plateau are associated with increased accumulation of ^{54}Fe compared to nearby Fe-poor layers, suggesting the Fe may have undergone redox cycling within estuarine or shelf environments prior to delivery to the Yermak Plateau."

L213: recommend "due to flocculation and sediment at low salinities"

We agree and this change has been made.

L218-220: As above, I'm not sure I would overrely on the Fe isotope data for this kind of interpretation (which takes a bit of a leap of faith). The differences are relatively small, and there could be other influences such as bedrock, or processing in subglacial systems.

We agree with this concern and have attempted to clarify (as described above) that this is a piece of evidence to support our model rather than something that we are overrelying on.

L220-222: How much of the shelf would be exposed at these points in time? And would glaciers discharge into fjords first, or directly off the shelf? In either case this interpretation of benthic iron shuttling might need some more thought or clarification in the text.

This is difficult to constrain, particularly beyond the most recent Fe-deposition event. We have attempted to clarify throughout the text that material likely had to be required in an estuary or shelf environment first, due largely to the scale of ascorbate extractable Fe enrichments and that this deposition and remobilization two-step process appears to have occurred only during intervals of expansion or retreat of glacial systems.

L262-264: This might also need some additional thought. There are still many tidewater glaciers in Svalbard, so this doesn't quite explain it all. Is it more the emergence of fjords as sediment traps or elemental sinks? I.e. the glaciers need to terminate outside of fjord systems onto the shelf or (in some instances) off the shelf.

We agree and have clarified that it isn't the retreat of tidewater glaciers alone, but specifically retreat away from the shelf and into the estuaries.

L293: More details needed here. What type of AAS? Precision/accuracy

We agree and more details have been added.

L299: How was P measured?

Details have been added.

L300-301: Is the HCl extraction data included in the manuscript?

No. This was an error that has been corrected.

References cited:

Hawkings, J.R., Wadham, J.L., Tranter, M., Raiswell, R., Benning, L.G., Statham, P.J., Tedstone, A., Nienow, P., Lee, K., Telling, J., 2014. Ice sheets as a significant source of highly reactive nanoparticulate iron to the oceans. Nat. Commun. 5. https://doi.org/10.1038/ncomms4929

Hopwood, M.J., Cantoni, C., Clarke, J.S., Cozzi, S., Achterberg, E.P., 2017. The heterogeneous nature of Fe delivery from melting icebergs. Geochemical Perspectives Letters 3, 200–209. https://doi.org/10.7185/geochemlet.1723

Reviewer #2

My main concerns are

- Although the authors present a nice dataset, the paper narrative needs work to be as accessible and clear/consistent as possible. There are several grammatical errors and sentences in need of rephrasing. The paper would benefit from some editing (some text is not needed) to be more precise and concise, tackling only what is most relevant to the central hypotheses of the paper.

We have address all grammatical changes and unclear sentences pointed out by the reviewer.

- There is a lack of error propagation and reproducibility in results that is not in line with the journal requirements. Particularly, Fe sediment extractions are notoriously accompanied by relatively high uncertainty, and so authors must state the reproducibility of results from each technique for this material to be of publishable standard.

Details on errors have been added.

- The paper discusses the reactivity of deposited sediments, and connects reactivity with bioavailability. However, there are several significant assumptions made here that are not clearly addressed, such as the post-depositional changes to Fe reactivity, the link between reactivity and bioavailability which is not tested, and the lack of general evidence that some 'reactive' Fe is actually bioavailable e.g. nano-goethite. The broader body of evidence that certain nanoparticles (Fe(II)-rich glaciogenic sediments, ferrihydrite) are bioavailable also remains slim (albeit growing) and the authors don't reference all the key papers! This hypothesis has not, to my knowledge, been tested on deep marine sediments. I recommend that the authors need to carefully define what they mean by 'reactive' and 'bioavailable', and discuss the post-depositional diagenesis that may have occurred

We have clarified our usage of the word reactive and softened the language surrounding "bioavailability. There is a paragraph on diagenesis and the assessment of its impact.

- The two methods – Fe sediment leaches and Mossbauer spectroscopy - used to determine iron phase speciation and reactivity - do not give the same results, but the evaluation of this feels incomplete.

Text has been added throughout the discussion to clarify that the pools measured by the two techniques are not the same but rather provide complementary information. The situations where the techniques disagree have been highlighted and discussed.

- I'm not overly convinced the Fe isotope dataset adds much, and the authors also describe in the methods a data subset of Fe isotopes (acid-leached pool) that is not presented in the results/figures

We agree and have updated the Fe isotope discussion (details above).

Abstract – haven't defined Fe as iron. Either keep as 'iron' in abstract and define in intro, or define Fe on line 21.

This was fixed.

Line 30 – I think this claim has too many assumptions. It suggests that all reactive Fe in sediments is bioavailable. Only certain reactive Fe forms have been shown to be somewhat available to phytoplankton, and this hasn't been tested on these deep marine sediments (or similar elsewhere). Additionally there is the matter of post-deposition changes to reactivity. I suggest that you adjust your language to say that some of the iron deposited may have been somewhat bioavailable at the time of deposition, and be careful throughout the paper in this interpretation since the sediment bioavailability has not been tested.

We agree and have updated this sentence to include fewer assumptions.

Line 47 – iceberg is one word

This was fixed.

Line 55 – not sure if this is called into question as even if loss 80 – 98 % of dissolved Fe, the remaining can still be biologically important, and plenty of papers show that meltwater delivers biologically important dFe (and particulate Fe) to the surface ocean. So despite the salinity gradient, Fe delivery to the marine system from glacial sources is shown to prevail. Several other studies link benthic supply of Fe from high latitude shelf sediments to increased primary productivity. Also, this paper discusses particulate Fe really, so what about this? Particularly since you suggest certain particulate Fe is bioavailable.

We updated this sentence to clarify.

Line 57 – 61 needs work. By southern high-latitude oceans – do you mean the Southern Ocean? Large regions of the Southern Ocean are critically light limited in winter as well, whilst large regions of the Arctic are nitrate limited during summer. You need a reference for Southern Ocean iron limitation. Browning and Moore (2023) Nat Comms, and Moore et al (2013) Nature look at global nutrient limitation so both of these would be appropriate to

show patterns of nutrient limitation in both polar regions. Generally, are you talking about present day or over glacial/interglacials as this is not clear? The focus of the paper is the last 135ka so make it clear to the reader.

The text has been updated for clarity and to include references.

Line 63 – 'nitrate-limited regions are largely controlled by iron supply' – this doesn't make sense. You mean the productivity in these regions is controlled by iron supply?

We agree that this point could be clearer. The following text was added "because Fe is required for the nitrogenase enzyme used by diazatrophs to fix nitrogen."

Line 66 – would be nice to have some melt/retreat rates included here.

Since we do not compare to retreat and melt rates in the past, we do not think this addition is needed.

Line 73 – 'biogeochemically reactive Fe'. This is the only time you use this phrase and there is no explanation of what is meant by this. I think this is really important to define what you mean when you say reactive early on, since the audience is quite broad. Are you talking about material that readily chemically dissolves in seawater, or material that is bioavailable? These are two distinct processes and are important for your focus on particulate/dissolved Fe and the link to the sediments.

We eliminated "biogeochemically" and added a sentence to define reactive Fe.

Line 85 – starting the results by describing the age model feels clunky. To a broad audience this isn't that clear either – perhaps most of this could be moved to the methods? Iron is the central tenet to this paper so put this up front.

We agree that the start to the results was clunky. So a later sentence was moved up and reorganized "To investigate changes in biogeochemically reactive iron since the last interglacial, we focus on the upper 6 m of core PS92/39-2, which covers the last 130 ka." However, we do still think it is important to start a paleoceanographic reconstruction with the age model before other geochemical results as it provides the backbone of the record.

Line 92 – perhaps put 'see methods' rather than listing undefined extraction methods that are probably unknown to much of a broad audience.

Since multiple extraction methods were used, the details are needed here. While we acknowledge that a broad audience might not be concerned, the community that investigates Fe cycling in marine and glacial systems will consider these details important, as evidenced by the request for more clarity about extractions from Reviewer 1. However, we changed the language to focus on the phases rather than the extractions, which will be more relevant to a larger audience.

Line 93 – can you put this in context? As that's a really high reactive Fe content but that's not clear here.

We agree that this is an important point, so we added context about the sequential Fe and ascorbate contents in the discussion.

Line 98 – quantitatively define 'elevated' FeT contents relative to your background

This has been updated to add (>5 wt%), which proves comparison to the background content.

Line 100 – 'based on modelling of Mossbauer results', 'ordered fe-oxyhydroxide… are reactive'. This is too vague. I think again you need to explain the implications of a reactive Fe pool and what's considered biologically important if that's what you're invoking.

We acknowledge that our description of the Mössbauer results and their interpretation in the main text was very – probably too – brief. We expanded on our results in the text and clarified what we consider to be reactive minerals: poorly ordered / incompletely ordered phases of small particle size.

We adapted the text in lines:

Based on Mössbauer spectroscopy, Fe (oxyhydr)oxides increase in samples with elevated FeT contents (51-62% of the Fe pool, as compared to 4-23%; yellow bars in Figure 3). This component exhibits Mössbauer parameters (Table S1) consistent with ferrihydrite and/or

goethite, with the latter being poorly ordered and/or of small size as indicated by its magnetic ordering occurring well below room temperature (reference: Thompson et al, 2025). We consider these poorly ordered Fe (oxyhydr)oxide(s), together with the incompletely ordered component we observed at 4 K (white bars in Figure 3), as reactive Fe phases.

Line 103 – this isotope value is a little abrupt. Has this been previously defined for reader.

This has changed due to other changes in the text.

Line 105 – 'P(T) tracks total Fe(T)' – this is too vague. Is there a statistically significant correlation?

We agree that this language is not useful and have updated the text with a quantitative observation.

Line 126 – comma after front.

This sentence has been changed.

Line 132 – comma after diverse

A comma has been added.

Line 136 – 'delivers pink sediments' – what does this mean?

This text has been updated to "pink-coloured" sediments.

Line 140 – 'the presence of tidewater…' this is vague and you don't discuss the implications of this.

This has been updated to clarify "because biogeochemically reactive iron generated within the subglacial environment can directly enter marine waters and not be trapped on land."

Line 146 – Yoshida et al. don't include goethite in their culturing experiments. Goethite is a more crystalline Fe (oxyhydr)oxide than the feed stock they use. They age pure ferric iron stock in seawater over 1 day to 3 weeks – defining this as amorphous Fe (oxhydr)oxides. I think there's some confusion here between these definitions, and to my knowledge there

has been no culturing study that shows goethite as bioavailable. See also Wyatt et al (2024) PNAS for culturing experiments with glaciogenic material, and Jones et al. (2025) Nature Communications for determining particulate Fe speciation delivered by glacial meltwaters.

This is a typo that has been corrected.

Line 164-165 – do you mean 370 cm?

This typo has been corrected.

Line 169 – this sentence and the following don't make sense and have grammatical errors.

Due to changes in the text, this sentence was eliminated.

Line 174 – I am not an Fe isotope expert but this analysis feels a little incomplete.

We agree. Due to space limitations, we have moved the isotopes to the supplemental to avoid over interpretation of small signals.

L:ine 186 – comma after water column. Periodic input of increased reactive Fe? May act to trap P, rather than prevented the flux of P, perhaps. There may be interaction and redissolution of these minerals back into the water column, so is 'prevented the flux of P' totally accurate?

These changes have been made.

Line 190 – glaciers are key generators of amorphous Si and glacial Si export is dominated by amorphous silica, so these peaks could also be interpreted as spikes in glacial Si delivery e.g. Hatton et al., 2019 (Proceedings of the Royal Society A).

While glacial Si export has been documented from meltwaters, to our knowledge, the transport of amorphous Si to open waters (~300 km offshore) has not been documented nor have significant deposition of glacially derived amorphous Si within seafloor sediments. With such a large sedimentary contribution of amorphous Si (up to >9 wt.%) compared to most

sediments, which did not contain measurable amorphous Si, biogenic Si associated with blooms is the most likely source.

Line 205 – Studies linking particulate Fe delivery by meltwater are relevant… Suggest Jones et al. (2025) Nature Communications, in which they directly measure particulate Fe and Fe speciation, finding a prevalence of Fe(II) and mixed valence Fe delivery by glacial meltwater to the surface ocean.

We agree that this is a valuable reference with significant relevance to the work and have added it.

Line 210 – unclear phrase. Has Fe mineral deposition been proposed as a proxy, or meltwater?

Yes. We replaced "it" with "Fe (oxyhydr)oxides to remove confusion.

Line 260 – 'delivery of reactive Fe significantly offshore' – what does this mean? That the Fe travels a large distance? I don't think this is the right wording.

We agree and updated this sentence to be clearer.

Line 263 – 'Fe delivery does not occur during the Holocene' – Don't you mean that Fe delivery is not recorded in the records at your site?

We have added "at any of our sites" after Holocene to clarify.

Line 267 – the final two references feel a bit out of place / poorly ordered. Suggest incorporating with the previous statement.

We agree and have updated this sentence.

Methods

Line 287 – were sediments freeze dried? This has been shown to change the mineralogy and reactivity of iron (oxhydr)oxides (Raiswell et al. 2010). No details of replicates or reproducibility / error propagation are provided.

The text already included the details about freeze drying. Freeze drying can decrease the reactivity of Fe phases, which means that our results show the minimum degree of reactivity and all samples were likely impacted in a similar way, indicating that this does not impact the conclusions of the paper.

Details about reproducibility and errors have been added.

Line 300 – what was the reproducibility of isotope measurements? Did you run any replicates? This needs stating and the error bars provided in Figure 3. Do you present the 0.5 M 1 hr HCl isotopes as I don't see them in your main or supplementary figures or results, but it would be helpful to have these data. Also, why is this sediment extraction protocol different to the Fe reactive phase 1M 0.5 hour extraction? This therefore represents a different Fe pool.

Errors have been added to the figures. The inclusion of details for the acid extraction was in error, as these were previously removed from the paper. It was excluded due to concern weak acid extractions cause fractionations in the Fe isotopes. We agree that the difference in extraction between concentrations and isotopes was also an issue.

Line 322 – can you explain why the Mossbauer data was not accompanied by standards? Considering the discrepancy between this and the Fe extractions, some discussion of error propagation of both of these methods is warranted. Does the Voigt-based fitting routine use built in standards for spectral analysis or were standards ran by you?

It is uncommon to run standards as part of Mössbauer measurements, as the fitting of the data is carried out via the hyperfine interaction parameters center shift, quadrupole split, and hyperfine field. These parameters are derived from a Hamiltonian and are known for standard materials / have been published in peer-reviewed articles and reviews (e.g, Dyar et al, 2006; Thompson et al, 2025) and reference books. In contrast to some other spectroscopic methods, Mössbauer spectral fitting does not rely on linear combination fitting of (ideal) standards.

The Voigt-based fitting routine (Rancourt & Ping, 1991) takes into account a distribution of quadrupole splittings that reflect "local and long-range structural and chemical environments acting concomitantly", which is typically found in natural samples. The fitting routine applies a least-square fit algorithm and produces a 'goodness-of-fit' factor, χ^2, which ideally should achieve a value close to 1. These values are provided in Table S1 for all fits, along with the standard deviations of the fitted spectral areas. These

standandard deviations were included in the error propagation to calculate the absolute content of each Fe component as shown in Figure 3B.

Line 329 – as above, for XRD. Did you measure any standards to verify?

Individual runs of XRD analysis are not run with standards. However, the method has been calibrated using standard measurements. In the methods paper Vogt et al. 2002, which is cited here the program QUAX was checked by standard measurements of two phase mixtures up to six phase mixtures and the quantification quality is fully reported. In the original setup of the reference mineral database of QUAX (see citations in Vogt et al. 2002) the standards used have been checked for pureness by elemental analysis, so the different Fe-mineral phases in the program have all been checked for their exact elemental composition.

Figures/tables

Figure 3 – Add error bars/comment on errors in caption, for 3a and 3c.

Error bars and discussion of errors have been added to figures.
